# Factors Associated with Physician Assistant Practice in Rural and Primary Care in Utah

**DOI:** 10.1155/2011/879036

**Published:** 2011-08-03

**Authors:** Jennifer M. Coombs, Perri Morgan, Donald M. Pedersen, Sri Koduri, Stephen C. Alder

**Affiliations:** ^1^Physician Assistant Program, The University of Utah, 375 Chipeta Way, Suite A, Salt Lake City, UT 84108, USA; ^2^Physician Assistant Program, DUMC 104780, Duke University Medical Center, Durham, NC 27710, USA; ^3^Utah Medical Education Council, 230 South 500 East, Suite 210, Salt Lake City, UT 84102-2062, USA; ^4^Division of Public Health, The University of Utah, 375 Chipeta Way, Suite A, Salt Lake City, UT 84108, USA

## Abstract

Physician Assistants (PAs) have become an integral part of the United States (U.S.) health care system since the profession began in the late 1960s. PAs have been suggested as solutions to predicted physician shortages especially in primary care. This study examined the predictors of primary care and rural practice patterns of PAs in Utah. A cross sectional survey design was utilized. The outcome variables were practice specialty and practice location. The predictor variables were age, gender, number of years in practice, location of upbringing, and professional school of graduation. There was a response rate of 67.7%. The Utah Division of Occupational and Professional Licensing (DOPL) provided the list of licensed PAs in the state. Physician assistants who reported being raised in rural communities were 2.29 times more likely to be practicing in rural communities (95% CI 0.89–5.85). Female PAs had lower odds of practicing in a rural area (OR: 0.26; 95% CI: 0.10–0.66). Female PAs had lower odds of practicing in primary care versus their male counterparts (OR: 0.56; 95% CI: 0.33–0.96). Graduation from the Utah PA Program was more likely to result in primary care practice (OR: 2.16; 95% CI: 1.34–3.49).

## 1. Introduction

Approximately 80% of the Utah population lives on the Wasatch Front, centering on Salt Lake City. Population growth along the Wasatch Front has made Utah one of the most urban states in the nation [1]. When the ratio of primary care physicians to population is assessed, Utah ranks last in the country [2]. There were 89.6 primary care physicians per 100,000 population in the US in 2008; Massachusetts ranked the highest with 129.4 primary care physicians and Utah the lowest, 63.4. Nationally, primary care shortages are predicted as increasing numbers of physicians are selecting specialty practice [3–7]. One strategy to address primary care shortages has been to turn to physician assistants (PAs). PAs have been shown to improve access to care for the underserved [8]. Increasing numbers of PAs are also entering specialty care although the flexibility and career mobility of PAs suggest they can also move out of specialties and into primary care [9]. 

A Utah study in 2006 found there were 4,484 total physicians, which equated to 165 physicians per 100,000 population. In 2003, 29% of Utah physicians were in generalist fields. This study estimated Utah would need to recruit up to 270 physicians per year due to population growth, age demographics, loss of full-time equivalent (FTE), and retirement. Out of state, trained physicians will be required because Utah's medical school will only meet 19%–22% of the projected annual demand for physicians [10]. Because the Governor's Office of Planning and Budget (GOPB) projects Utah's overall population to increase from 2.7 million to 3 million by 2012 and another million by 2020, more doctors may be needed. According to the US Census Bureau, Utah was the second fastest growing state in the nation during 2009 with an annual growth rate of 2.1%. The number of Utahans over age 65 (as a percentage of the population) is expected to increase with estimates that the age 65 and older population will grow from 213,201 in 2000 to 319,564 in 2015 (a growth rate of 50%) [11]. 

The demographics of Utah are unique, ranking first in the country for population growth (due almost entirely to a high fertility rate) [12]. More than 20% of the population are dependent children, stretching public services including public schools and health care [13]. Three factors—high fertility rate, growing elderly, and increased utilization of health care services—are expected to increase the demand for primary care services.

Since the turn of the new century, workforce studies have focused on the increasing supply of PAs in the state. In Utah, the visibility of PAs has been increasing. From 2003 to 2008, the number of PAs has grown from 324 active PA licenses to 700 active PA licenses (116% growth). 

To better understand the current distribution of PAs, along with the enablers and barriers to primary care and rural practice selection, we undertook a study on this labor force. Our research questions center on the following.

1. What is the distribution of PAs in the state of Utah?
2. What are the factors associated with primary care and rural practice selection?
3. Can PAs help address the increasing need for primary care providers in Utah by choosing primary care specialties?

## 2. Methods

A list of licensed PAs was obtained through the Utah Department of Commerce's Division of Professional Licensing (DOPL). In 2008, there were 700 licensed PAs in the state; a survey was sent to every PA. A total of three separate mailings were conducted over the period of October 2008 through January 2009. A total of 474 responses were received which equates to a 67.7% response rate. Of the 474 respondents, 432 PAs said they are actively practicing in Utah (90.7%). Survey responses were not weighted for nonrespondents for the purpose of the regression analysis. When checked for potential response biases based on year of licensure, age, gender, and geography, a particularly low response rate was identified for Iron County, a rural county located in southwest Utah. Targeted mailings to the PAs with addresses located in Iron County were conducted in an attempt to increase the response rate from that county. 

### 2.1. Design of the Survey

A survey instrument was constructed based on two previous surveys (1998 and 2003) and a physician workforce survey (2006). A PA study committee included the University of Utah PA Program Director, the Executive Director of the Utah Academy of Physician Assistants, and three practicing PAs. Useful questions from previous surveys were incorporated.

### 2.2. Study Population

The study population was the universe of PAs licensed to practice in Utah in October 2008. PAs were categorized as primary care if their self-reported specialty was family practice, general internal medicine, pediatrics, or obstetrics and gynecology. Rural or urban designation was based on county. Cache, Davis, Provo, Salt Lake, Weber, and Washington County were considered urban; 20 of the 26 counties were classified as rural. Utah has 5 urban communities, Salt Lake, Logan, Ogden, Provo, and St. George and within these so-called urban communities, rural communities exist. The remaining counties all have populations less than 50,000 in the county.

### 2.3. Study Variables and Statistical Analysis

Five variables related to demographic information were available within the survey. Multinomial logistic regression analysis was performed using SPSS 16.0 to assess the relationship of the predictive variable to the outcome of rural and/or primary care practice, using odds ratios with 95% confidence intervals (CIs). 

## 3. Results

Responses were obtained from 432 of 700 physician assistants who are actively practicing in the state of Utah (Table 1). Thirty-six percent of Utah PAs are between the ages of 31 and 40 years, with males at 60.6% of the total respondents. Nearly half (47.4%) of the respondents have been in practice between 0 and 5 years. Location of upbringing was 17.2% urban, 52.2% suburban, and 30.5% rural. Nearly half (47.6%) of the respondents graduated from the University of Utah Physician Assistant Program. Forty-five percent of physician assistants in Utah provide primary care and 85.3% practice in an urban location.

Except for Washington County, all of the counties with greater than 40% nonresponse rate were counties with fewer than 10 PAs in them. The only county with more than 5 PAs and greater than 50% nonresponse rate was Iron County. A targeted separate mailing was sent to Iron County PAs in order to attempt to increase response rates (Table 2). Response rate for males was higher than females, 71% and 63%, respectively. Increasing age and years of license resulted in slightly higher response rates (Table 3).

In logistic regression analysis, PAs age 31–40 had the highest odds of practicing in primary care (OR: 1.74; 95% CI: 0.85–3.57); however, it did not achieve statistical significance (Table 4). Female PAs had lower odds of practicing in primary care versus their male counterparts (OR: 0.56; 95% CI: 0.33–0.96). PAs had lower odds of practicing primary care if they reported a rural or suburban upbringing (OR 0.49; 95% CI: 0.26–0.93, and OR: 0.33; 95% CI: 0.16–0.66). Graduation from the Utah PA Program was more likely to result in primary care practice (OR 2.16; 95% CI: 1.34–3.49). The only statistically significant predictors of primary care practice were being male (*P* = 0.036), obtaining training in the state of Utah (*P* = 0.002), and urban upbringing (*P* = 0.008).

In logistic regression analysis, female PAs had lower odds of practicing in a rural area (OR: 0.26; 95% CI: 0.10–0.66, *P* = 0.005) (Table 5). PAs who reported graduating from the Utah Physician Assistant Program had higher odds of practicing in a rural area (OR: 1.33; 95% CI: 0.67–2.65, *P* = 0.413), but this did not achieve statistical significance. PAs who practiced in a rural environment were more likely to report a rural upbringing (OR: 2.29; 95% CI: 0.89–5.85, *P* = 0.001). The only statistically significant factors were male gender (*P* = 0.005) and rural upbringing (*P* = 0.001). Age and years of practice were not significantly associated with predictors of rural practice in Utah. From 2003 to 2008, specialty practice choice of family medicine declined from 40% to 31.5% of the total Utah PA workforce (Table 6).

## 4. Discussion

PAs in Utah mirror the changing workforce demographics of the state: young, primarily urban, and suburban-raised professionals. Under the current scenario, the potential use of PAs to blunt predicted rural and primary care shortages of Utah physicians may fall short. Within the last 10 years, Utah has increasingly relied on PAs trained in other states because the state's PA program of 40 PAs graduates per year is insufficient. In 2008, a total of 88 PAs practiced in rural counties, consisting of 21.6% (19) female PAs and 78.4% (69) male PAs. 

Efforts are underway to bolster the nation's primary care workforce, and the Patient Protection Affordable Care Act (PPACA) legislation of 2010 injects $250 million to improve primary care education for doctors, PAs and NPs. However, primary care may not be possible without incentives to practice in this specialty because salary was not independently predictive of either rural or specialty practice, nor were years of practice [9]. Influencing factors upon primary care and/or rural practice include loan repayment and tax incentives—strategies that have been employed with success in other states. 

Although many PAs have been shifting into specialty practice, primary care still remains a viable choice for many PAs entering the workforce [14]. The finding that female PAs may be more likely to practice in specialty care may be due to the increased job availability of specialty care in recent years. PAs may be different from their physician counter parts when it comes to specialty choices [15]. Utah PAs were twice as likely to practice in a rural environment if they reported being raised in a rural community. Male gender has been significantly associated with rural practice and is reflected in this research. Although Utah has been traditionally male dominated PA profession, this is slowly changing. On average, the PA workforce in Utah graduated from a PA training program 15 years ago (median of 8 years). The mean number of years of experience for male PAs is much higher than for females PAs. There are 21.3% (79) of male PAs in the Utah workforce with over 20 years of experience, whereas only 6.1% (15) of female PAs have greater than 20 years of experience. 

In this study, rural location of upbringing was associated with statistically significant lower odds of practicing in primary care when compared to urban location of upbringing. One possible explanation is the overall increase in urban location of many primary care practice jobs. In general, the results of this study showed increased urbanization of the young profession. This may explain why urban upbringing is a predictor of primary care practice.

Limitations of this study include the 67.7% response rate to the survey. This is higher than the American Academy of Physician Assistants (AAPA) survey 2009 response rate of 35%. The data were not weighted in the analysis. Low numbers of PAs practicing in some rural areas may limit the analysis. Effort was made to locate the PA practice location, not the home address in the analysis, and zip codes were used to classify county of practice. Because Utah has a clear pocket of urban living on the western slope of the Wasatch Mountains and one more pocket in the southern end of the state, and given the small number of PAs in the state, finer zip code detail in the analysis did not result in a more informative study. 

A second limitation of the study was the self-classification of rural, urban, and suburban upbringing. Because of the changing nature of rurality in the state and the age of the person answering the question, it was decided that self-classification was the method to employ. In this case, perception may be reality, and primarily for the sake of simplicity, this method, which has been employed in other similar studies, was utilized [16]. Finally, the classification of rural, urban, and suburban was used in the survey to improve the self-reporting of upbringing, although the analysis only used rural or urban in reporting outcomes.

## 5. Conclusion

Factors such as rural, urban, or suburban upbringing, gender, age, and years of practice are important as they relate to primary care and rural health care practice among PAs in Utah. A consistent and well-trained supply of PAs is critical to access to care for Utah citizens. Our findings suggest Utah will continue to experience shortages of primary care physicians that will be amplified in underserved and rural communities. Substantially increasing the number of PAs practicing in these areas may require a number of strategies that take into consideration demographic as well as personal factors. Rural versus urban practice choice among PAs in Utah could potentially be influenced by recruitment, training, and retention efforts that facilitate workforce placement in critical areas. Key groups and leaders in primary care and rural health care could be canvassed as to how to implement effective strategies to influence PAs to enter primary care and/or rural practice. For example, county commissioners, small town majors, rural hospital administrators, and local health department employees may have special interests and expertise in PA recruitment and mentoring. An absence of proactive strategies may be an opportunity missed as the path toward increasing specialization and urbanization has been well worn.

## Figures and Tables

**Table 1:** 2008 Utah PA characteristics for the study population.

PA characteristics	*N* = 432	Percent
Age		
21–30	55	12.7%
31–40	158	36.6%
41–50	100	23.1%
51–60	91	21.1%
61+	28	6.5%
Gender		
Female	170	39.4%
Male	262	60.6%
Years of practice		
0–5	203	47.4%
6–10	89	20.8%
11–15	67	15.6%
16–20	34	7.9%
21+	35	8.3%
Practice type		
Primary care^1^	191	45.6%
Specialty care	228	54.4%
Practice location^2^		
Practice in rural county	60	14.7%
Practice in urban county	349	85.3%
Location of upbringing^3^		
Urban	74	17.2%
Suburban	224	52.2%
Rural	131	30.5%
Physician assistant school of graduation		
Utah	203	47.6%
Other	223	52.3%

*474 surveys were returned, 432 (90.7%) reported they are clinically active in Utah.

^1^Primary care definition: family practice, general internal medicine, pediatrics, and obstetrics and gynecology.

^2^Five Utah counties, Cache, Provo, Salt Lake, Weber and Washington county: urban; all others are considered rural. Location based on practice address zip code.

^3^Self-reported location of upbringing rural/suburban/urban.

**Table 2:** Response characteristics by county.

County	Response rate
Beaver	50.0%
Box Elder	60.0%
**Cache^1^**	78.3%
Carbon	50.0%
Daggett	100.0%
**Davis**	63.1%
Duchesne	33.3%
Emery	100.0%
Garfield	0.0%
Grand	66.7%
Iron	44.4%
Juab	100.0%
Kane	100.0%
Millard	100.0%
Morgan	0.0%
Rich	100.0%
**Salt Lake**	70.6%
San Juan	100.0%
Sanpete	60.0%
Sevier	100.0%
Summit	66.7%
Tooele	62.5%
Uintah	100.0%
**Utah**	68.0%
Wasatch	50.0%
**Washington**	56.4%
Wayne	100.0%
**Weber**	66.0%
Out of State	58.9%
Total	66.7%

^1^Bold: US Census Bureau metropolitan area designation (urban county).

**Table 3:** Response rate by age, gender, and years of license.

Demographic variable	Response rate
Gender	
Male	71%
Female	63%
Age	
21–30	66.6%
31–40	64.6%
41–50	64.1%
51–60	71.8%
61+	71.4%
Years of licensure	
0 to 5	66.0%
6 to 10	67.4%
11 to 15	65.1%
16 to 20	75.0%
21+	77.7%

**Table 4:** Predictors of primary care^1^/specialty care practice.

Independent variable	*N* = 381*	OR (95% CI)	*P* value
Age ^a^			
21–30	53	1.00	—
31–40	137	1.74 (0.85–3.57)	0.129
41–50	87	1.58 (0.67–3.74)	0.289
51–60	83	1.49 (0.55–4.02)	0.429
61+	21	0.52 (0.12–2.15)	0.367
Gender ^b^			
Male	227	1.00	—
Female	154	0.56 (0.33–0.96)	0.035
Years of practice ^2,c^			
0–5	183	1.00	—
6–10	77	0.67 (0.35–1.28)	0.228
11–15	60	0.33 (0.14–0.76)	0.010
16–20	32	0.26 (0.09–0.76)	0.014
21+	29	0.49 (0.15–1.51)	0.214
Physician assistant school of graduation ^d^			
Non-Utah PA school	198	1.00	—
Utah	183	2.16 (1.34–3.49)	0.002
Location of upbringing ^3,e^			
Urban	66	1.00	—
Suburban	198	0.49 (0.26–0.93)	0.029
Rural	117	0.33 (0.16–0.66)	0.002

*After deleting missing cases for all predictor variables listed above (for logistic regression), 381 of the 432 total records were left for analysis.

^1^Primary care definition: Family practice, General internal medicine, pediatrics, and obstetrics and gynecology.

^2^Years of practice: number of years since first license issue.

^3^Self-reported location of upbringing rural/suburban/urban.

Baseline category for comparison is:  ^a^age <31 yrs;  ^b^male;  ^c^0–5 yrs of practice;  ^d^non-Utah PA school graduate;  ^e^urban upbringing.

**Table 5:** Predictors of rural/urban^1^ practice.

Independent variable	*N* = 381*	OR (95% CI)	*P* value
Age ^a^			
21–30	51	1.00	—
31–40	136	0.98 (0.33–2.92)	1.012
41–50	86	1.18 (0.34–4.09)	0.848
51–60	76	1.48 (0.34–6.35)	0.674
61+	19	2.08 (0.31–14.0)	0.479
Gender ^b^			
Male	218	1.00	—
Female	150	0.26 (0.10–0.66)	0.005
Years of practice ^2,c^			
0–5	179	1.00	—
6–10	76	0.34 (0.12–0.97)	0.045
11–15	57	0.75 (0.24–2.29)	0.617
16–20	28	0.42 (0.09–1.87)	0.255
21+	28	0.21 (0.03–1.26)	0.089
Physician assistant school of graduation ^d^			
Non-Utah PA school	190	1.00	—
Utah	178	1.33 (0.67–2.65)	0.413
Location of upbringing ^3,e^			
Urban	62	1.00	—
Suburban	191	0.62 (0.23–1.67)	0.352
Rural	115	2.29 (0.89–5.85)	0.083

*After deleting missing cases for all predictor variables listed above (for logistic regression), 381 of the 432 total records were left for analysis.

^1^Five Utah counties, Cache, Provo, Salt Lake, Weber, and Washington county: urban; all others are considered rural. Location based on practice address zip code.

^2^Years of practice: number of years since first license issue.

^3^Self-reported location of upbringing rural/suburban/urban.

Baseline category for comparison is:  ^a^age <31 yrs;  ^b^male;  ^c^0–5 yrs of practice;  ^d^non-Utah PA school graduate;  ^e^urban upbringing.

**Table 6:** Comparison of specialty for practicing physician assistants in Utah, 2003 to 2008*.

Specialty	2003	2008	Change (percentage points)
Family medicine	40%	31.5%	−8.6
Orthopedic surgery	6.5%	10.6%	4.0
Emergency medicine	5.4%	6.0%	0.6
Pediatrics-General	6.2%	5.8%	−0.4
Internal Medicine/General	6.5%	4.4%	−2.2
Dermatology	4.2%	4.2%	0.0
Prev Med/Occ Med	3.8%	3.2%	−0.7
Urology	1.9%	3.0%	1.1
Cardiology	3.1%	2.8%	−0.3
Hematology/Oncology	2.7%	2.8%	0.1
OB/Gynecology	1.5%	2.5%	1.0
Other Surgical Subspecialty	0.8%	2.5%	1.7
Cardio-Thoracic Surgery	1.5%	1.9%	0.4
Otolaryngology	0.4%	1.9%	1.5
Neurology	1.2%	1.6%	0.4

*Includes specialties with 10 or more PAs.
